# Whole Grains Perspective: Porridge or Popcorn^☆^

**DOI:** 10.1016/j.tjnut.2026.101560

**Published:** 2026-04-28

**Authors:** Yuchen Jia, Victoria Miller, David JA Jenkins

**Affiliations:** 1 Food, Nutrition and Health Program, Faculty of Land and Food Systems, The University of British Columbia, Vancouver, British Columbia, Canada; 2 Department of Global Health, Population Health Research Institute, Hamilton, Ontario, Canada; 3 Department of Medicine, McMaster University, Hamilton, Ontario, Canada; 4 Departments of Nutritional Sciences and Medicine, Temerty Faculty of Medicine, University of Toronto, Toronto, Ontario, Canada; 5 Li Ka Shing Knowledge Institute, St. Michael’s Hospital, Toronto, Ontario, Canada

## Abstract

Terms such as “whole grains,” “fiber,” and “ultraprocessed food” are too broadly defined to guide specific disease-prevention strategies. More precise classification may be important for meaningful application in disease prevention. We wish to discuss the following 3 underexplored issues regarding whole grain consumption and noncommunicable disease (NCD) prevention: *1*) which foods are broadly recognized as “whole grains”; *2*) whether the apparent health benefits of whole grains reflect food choice itself or broader lifestyle choices of those who consume them; and *3*) whether specific types of whole grains confer distinct physiologic benefits. Of 12 major prospective cohorts (>100,000 participants), 10 assessed whole grain intakes, but only 3 whole grain foods (popcorn, oatmeal, and brown rice) were common to 7 of these cohorts, and 17 of 54 whole grain foods were listed by a single cohort only. This inconsistency highlights the need for standardized classification. Furthermore, some health benefits attributed to whole grain consumption may be partly explained by lifestyle characteristics of those who consume them. For example, whole grain consumers tend to smoke less, exercise more, and have higher levels of education, all of which independently reduce NCD risk. Finally, not all whole grains are equivalent, and the degree of processing matters. Intact whole barley, as consumed in barley stew, produces a lower postprandial glucose response (or glycemic index) than milled barley flour used in breads. Whole grains encompass a range of carbohydrate foods whose effects on NCD outcomes vary depending on whole grain type and processing. The physiological effects of different whole grain foods require exploration to promote more meaningful use for public health and clinical application. In cohort studies, despite covariate adjustment, some observed benefits may be due to the other lifestyle habits of those who select to be whole grain consumers.

Whole grain consumption is universally recommended in dietary guidelines to prevent and manage noncommunicable diseases (NCDs), particularly diabetes and cardiovascular disease (CVD). However, without a simple definition for what whole grains are, specific dietary advice is difficult. Further, there is an assumption that all whole grains fulfill similar roles. Finally, it is assumed that eating whole grains results in good health rather than that it is the healthy people who, for various reasons, prefer to eat whole grains. We therefore wish to address these issues by exploring the diversity of this category and the different physiological roles specific classes of whole grains may play in disease prevention.

## What are Whole Grains?

In a very broad definition, which may have little specific clinical value, whole grains are generally defined as a grain or grain product of any cereal or pseudocereal that contains the endosperm, germ, and bran layers, which should be present in the same relative proportions as in the intact kernel [[1], [2], [3]]. After inedible components such as the hull and husk are removed, it should consist of the intact, cracked, flaked, ground, or otherwise processed kernel. Whole grains from different sources, after milling or processing to differing degrees, may be consumed as such or added to other foods or dishes, depending on cultural preferences and traditions. Whole grain foods are consumed throughout the day in meal-specific contexts (e.g., oatmeal porridge at breakfast), and the method of processing may determine the physiological effects. However, national guidelines around the world define whole grains differently [1]. In 2021, Brazil’s National Health Regulatory Agency (Agência Nacional de Vigilância Sanitária – Anvisa) published a regulation for grain-based foods stating that a product can be labeled as whole grains if ≥30% of its ingredients by weight are whole grains, and the product contains more whole grain than refined grain ingredients by weight [4]. The United States Food and Drug Administration defines a whole grain as containing ≥50% of all 3 parts of the original kernel—bran, germ, and endosperm [5]. Even if the grain is cracked, crushed, flaked, or made into flour during the milling process, it is still considered a whole grain if these parts remain in the same relative proportions as in the intact grain. The development of international guidelines to harmonize definitions and ensure consistency in research, labeling, and dietary guidance has been led by organizations such as the Whole Grain Initiative, International/Cereals & Grains Association, and the Healthgrain Forum, which have published or endorsed unified global definitions and guidance documents [3,6,7].

**FIGURE 1:**
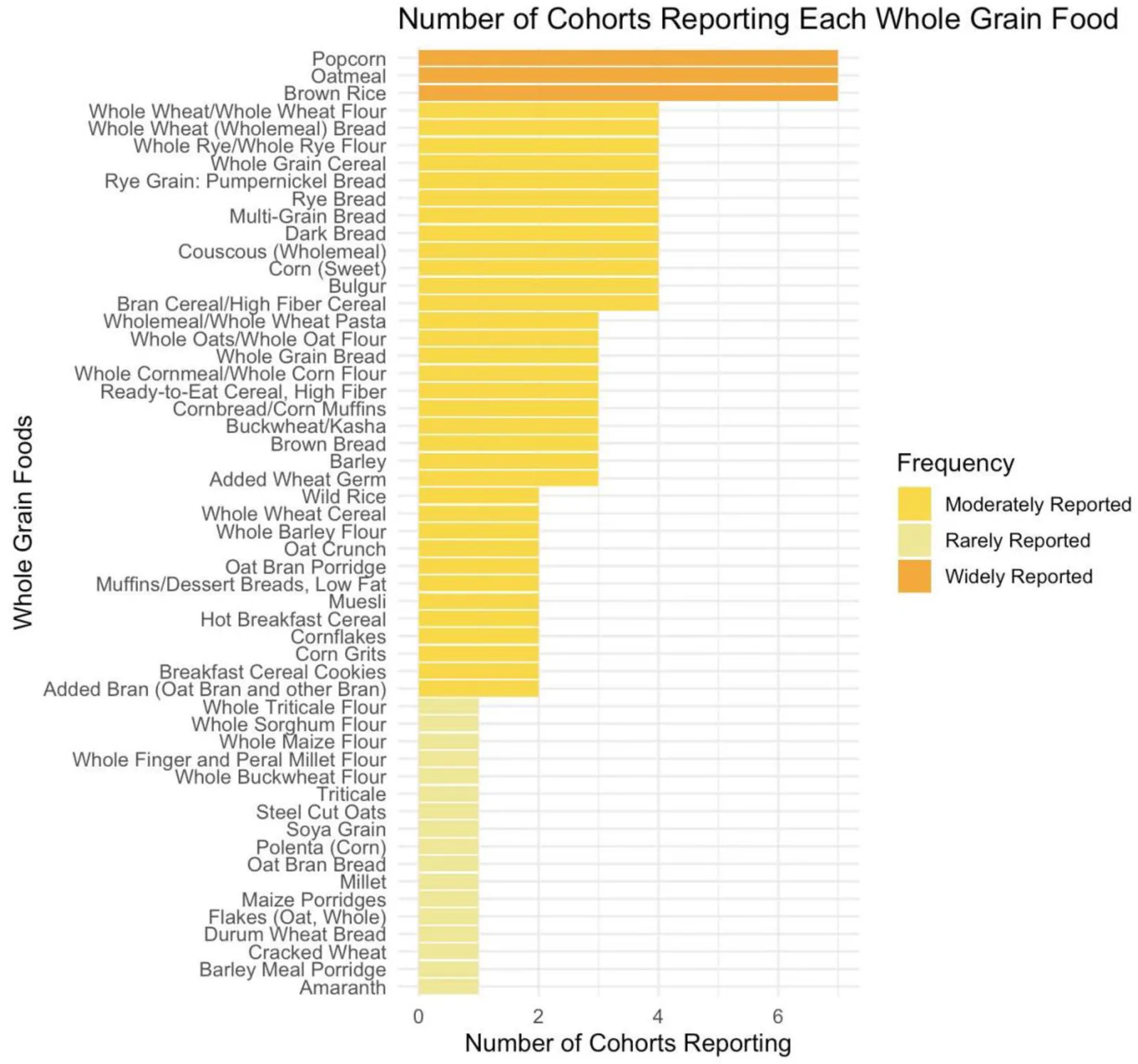
Bar chart showing the number of cohorts reporting each whole grain food in their food frequency questionnaire, where frequency is presented as widely reported, moderately reported, and rarely reported.

In the classification of whole grain foods, the whole grain ingredient must be differentiated from the whole grain food, information on the processing compared with the formulation should be provided, and the whole grain ingredient in a food should be expressed as the proportion of the daily gram weight. Lack of standardization remains part of the reason for the lack of adequate classification of whole grain food consumption. This complexity of the definition is well illustrated by the differing whole grains used by 12 of the largest prospective cohorts (>100,000 participants), largely from the Richard Doll Consortium, which also included data on glycemic index (GI)/glycemic load [8]. Among these cohorts, 10 assessed the association of whole grain consumption with a reduction in NCDs [[9], [10], [11], [12], [13], [14], [15], [16], [17], [18]]. The list of the specific foods each cohort defined as whole grains in their food frequency questionnaires that contributed to the term whole grains is shown in Figure 1. Each food was assigned a score based on the number of cohorts that classified the food as a whole grain, thus the score could potentially range from 1 to 10.

However, despite all cohorts claiming to be assessing whole grains, only 3 whole grain foods: oatmeal, brown rice, and popcorn, were common to 7 of the 10 cohorts, whereas 17 of the total of 54 whole grain foods were only used by one of the 10 cohorts (Figure 1). This lack of uniformity is not recognized by experts in meta-analysis who aim to inform public advice, but for whom whole grain is a single generic term, a term that may even obscure specific benefits of specific types of whole grain foods [19,20].

Studies of Kissock [21] have assessed the effects of Australian and Swedish prospective cohorts using both the healthy whole grain definition (30% whole grain dry weight) and a regular whole grain cohort food assessment. The association was poor, but nevertheless, assessments related to CVD in the cohorts indicated that a degree of variation in the whole grain food assessment did not prevent the demonstration of the association with disease outcomes.

## Other Ingredients

Whole grain foods may not consist of 100% whole grain. Although oatmeal is likely to be 100% oats, whole wheat foods may vary from 30% to >50% whole wheat depending on the jurisdiction. Furthermore, whole grain and non-whole grain products may be “enriched” with specific whole grain components to increase the physiological effects, for example, wheat bran to increase laxation and the perceived fiber benefits. Alternatively, semolina, a coarse flour from protein-rich durum wheat that provides many of the healthy advantages of whole wheat, but is classed as a white flour product to produce noodles, spaghetti, etc. Finally, whole grains contain minerals, vitamins, plant phenolics, and other “phytochemicals” (e.g., polyphenolics) which confer potentially important health benefits.

## Do “Healthy People” Prefer to Eat Whole Grains?

A further issue that complicates the association between whole grain consumption and freedom from NCDs is the question of causality. Specifically, does whole grain consumption reduce the risk of NCDs, or do healthier individuals, who are at a lower risk of NCDs, also tend to consume whole grains because they enjoy them, or consume them as a cultural habit or have been encouraged to do so by family, friends, or public health messaging? We have therefore looked at participants’ various lifestyle characteristics and pooled data across the 10 major cohorts where data were available to assess lifestyle patterns associated with the highest compared with the lowest quantile of whole grain intake. Specifically, we collected data, if available, for the association of whole grain consumption with non-diet-related health attributes, including smoking status (never smoked %); age (years); BMI (kg/m^2^); alcohol intake (g/d); physical activity (total MET-h/wk or total metabolic equivalent h/wk); education (college or postgraduate education %); and socioeconomic status. We expressed each lifestyle characteristic as the mean difference between the highest and the lowest quantiles of whole grain intake and pooled the individual quantile differences for each outcome. Comparisons were performed using the one-sample t-test to assess whether the mean differences differed from zero [22]. A two-sided *P* value of < 0.05 was considered statistically significant, and values were rounded to 3 decimal places (Table 1). Where data were available, higher whole grain consumption was significantly associated with nonsmoking status, older age, lower BMI, having more physical activity, and having a higher education level (*P* < 0.05 for all). These findings suggest that whole grain consumption may be part of a group of healthy lifestyle attributes that play a role in the prevention of NCDs. These associations persisted despite the studies’ attempts to adjust for lifestyle factors, although such analyses may be limited or incomplete. Whole grain consumption may simply be one of the larger groups of healthy personal attributes, or perhaps just a marker of healthy lifestyle attributes.

**TABLE 1:** Comparison of participants’ lifestyle characteristics between the highest and lowest quantiles of whole grain intake.

Participants’ characteristics^1^	Cohorts (*n*)	Average number of quantiles	Mean difference between highest and lowest quantiles (SE)	Significance *P* value
Smoke^2^ (never smoked %)	8	4.89	9.19 ± 2.10	0.002^7^
Age^2^ (years)	8	4.89	0.79 ± 0.32	0.041^7^
BMI^3^ (kg/m^2)^	5	5	−0.78 ± 0.15	0.007^7^
Alcohol^3^ (g/d)	5	5	−1.74 ± 0.87	0.116
Physical activity^4^ (total MET-h/wk)	4	5.25	6.40 ± 0.77	0.004^7^
Highest education^5^ (college or postgraduate %)	5	4.83	10.23 ± 1.49	0.001^7^
Low socioeconomic status^6^	2	5	−2.30 ± 0.50	0.136

1 Participants’ characteristics derived from publications on the very large cohorts (>100,000 participants).

2 Smoking status and age outcomes used the Cancer Prevention Study II (CPSII); National Institutes of Health–American Association of Retired Persons Diet and Health Study (NIH-AARP); Prostate, Lung, Colorectal, and Ovarian Cancer Screening Trial (PLCO); Women’s Health Initiative (WHI); Harvard Nurses’ Health Study I (Harvard NHS I); Harvard Nurses’ Health Study II (Harvard NHS II); Harvard Health Professionals Follow-Up Study (Harvard HPFS); and Prospective Urban Rural Epidemiology Study (PURE) cohorts.

3 Body mass index and alcohol consumption outcomes used PLCO, WHI, Harvard NHS I, Harvard NHS II, and Harvard HPFS cohorts.

4 Physical activity outcome used WHI, Harvard NHS I, Harvard NHS II, and Harvard HPFS cohorts.

5 Education outcome used CPSII, NIH-AARP, PLCO, WHI, and PURE cohorts.

6 Socioeconomic status outcome used PLCO and WHI cohorts in units of % unemployed or income below 35,000.

7 *P* < 0.05.

## Do Specific Whole Grains Have Specific Physiological Benefits?

Although there is debate over the definition of whole grains, there is almost uniform agreement on the overall health benefit of whole grain consumption [[9], [10], [11], [12], [13], [14], [15], [16], [17], [18]]. However, specific whole grains may confer specific large benefits and others very little or none. These major benefits may be obscured by the use of the collective term “whole grains,” which allows all whole grains, regardless of specific benefits, to share these beneficial characteristics. We have therefore explored whether specific whole grains may reduce postprandial glucose fluxes as an acute marker of improved glycemic control [23], which might relate to reduced risk for diabetes, CVD, and all-cause mortality. Previous studies have shown that major differences exist between intact or cracked whole grains and whole grain flour products, as well as their effects on the postprandial glycemic response [[24], [25], [26]]. For the same whole grain, we have therefore compared the glycemic responses, as GI, between the intact, crushed, or cracked whole grain foods, and milled flour products (Table 2) [27,28]. The mean GI differences and one-sample t-test were used to identify statistically significant differences between different forms of the same “whole grain” [26].

**TABLE 2:** The glycemic index difference between intact whole grain products and corresponding whole grain flour products.

Intact whole grain products	GI	Whole grain flour products	GI	GI difference
Rye grain: pumpernickel bread	49^2^	Rye bread	60^2^	11
Bulgur	47^2^	Whole wheat bread	72^1^	25
Steel cut oats	52^2^	Oat bread	65^2^	13
Barley	30^2^	Whole barley bread	72^2^	42
Whole corn	53^2^	Cornbread/corn muffins	74^2^	21
Buckwheat	51^1^	Buckwheat noodles	59^2^	8
Millet	71^1^	100% foxtail millet flour pancakes	76^2^	5
Mean	50.43	Mean	68.29	Mean 17.86 SE 4.82 *P* = 0.0101^3^

1 All glycemic index (GI) values were obtained from the study by David Jenkins et al. (1981).

2 All glycemic index (GI) values were obtained from the study by Fiona Atkinson et al. (2021).

3 *P* < 0.05.

As can be seen in Table 2, for all comparisons, the intact, cracked, or crushed whole grain products produced a reduced glycemic response (lower GI) compared with the whole grain flour products (*P* = 0.01). These data illustrated that when intact, cracked or crushed products are compared with whole grain flour foods (predominantly breads), reductions in postprandial glycemic responses are seen related to particle size and thus, by inference, their slower rate of digestion. Different forms of whole grains, therefore, appear to respond differently, and the generic term “whole grains” may obscure the value of specific types of whole grain that would be of use for specific disease-prevention strategies.

## Recommendations

There is a need to report whole grain intake in detail so that cohort evidence can be interpreted and compared with minimal measurement challenges and confounding. Perhaps of key importance is to report the qualification of whole grains as grams dry weight, and the definitions of whole grain used. Because of differences in the physiological effects of different whole grains, the type of whole grain assessed must be provided. Additionally, to predict physiological effects, the form in which the whole grain is consumed is important to record (intact, cracked, crushed, coarse, or fine milled). Food preparation is also of key importance in determining the physiological effect, in what form the whole grain is incorporated into the diet. These details will greatly enhance the ability to pool data on whole grain consumption from different trials and prospective cohorts meaningfully [29].

In summary, the “whole grain” terminology as a category requires refinement. At present, whole grains are widely recommended in dietary guidelines, such as the World Health Organization and American Heart Association, yet remain poorly defined and inconsistently applied, with benefits always seen for the class as a whole [18,19,25,[30], [31], [32]]. Our perspective highlights the need to distinguish between the physiological effects of specific whole grain types that may relate to the prevention of specific disease outcomes, and also the broader health benefits that may arise from generally healthy behaviors. Although whole grain consumption is linked to reduced glycemic responses, improvements in low-density lipoprotein, lower inflammatory C-reactive protein, and increased fecal bulk (laxation and reduced “straining at stool”), these benefits are broad and are related to specific types of whole grain and associated fibers [31,32]. Also, these benefits may be enhanced by other healthy lifestyle practices common among whole grain consumers. As a result, if whole grain consumption is to be recommended clinically and to the general population, it is important to determine which health effects are attributable to which specific types of whole grains. To strengthen the scientific and clinical value of whole grain recommendations, more precise terminology is required regarding the processing methods used, the specific types of whole grain (e.g., wheat, barley, oats, etc.), and the type of fibers associated with each grain, to reflect their distinct physiological effects [33]. Just as specific drug names are used to target specific chronic diseases and risk factors in pharmacologic treatments, the same specific definitions should be used for whole grains and specific diseases [34]. Greater clarity will help ensure that dietary guidance is evidence-based and optimized for both individuals with specific conditions and also the general population. Ultimately, refining this terminology may sustain interest in lifestyle-based strategies by demonstrating that precise dietary advice can meaningfully improve specific health outcomes.

## Author contributions

The authors’ responsibilities were as follows – DJAJ: posed the questions to be answered; YCJ, VM, DJAJ: responsible for the design, writing, and final content; and all authors: read and approved the final version.

## Funding

The authors reported no funding received for this study.

## Declaration of Generative AI and AI-assisted technologies in the writing process

AI and AI-assisted technologies were not used in the preparation of this work.

## Conflict of interest

DJAJ’s wife has previously been a director of INQUIS Clinical Research Ltd, which is a company that undertakes clinical studies and GI testing. All other authors report no conflicts of interest.
